# Measurement Invariance of the Satisfaction with Life Scale by Sexual Orientation

**DOI:** 10.1007/s10508-021-02240-0

**Published:** 2022-05-19

**Authors:** Irene Checa, Donatella Di Marco, M. Rocío Bohórquez

**Affiliations:** 1 Departamento de Metodología de Las Ciencias del Comportamiento., Universitat de València, Av. Blasco Ibáñez, 21., 46010 Valencia, Spain; 2 Departamento de Psicología Social, Universidad de Sevilla, C/Pirotecnia S/N, Seville, Spain; 3 ISCTE-IUL, Business Research Unit (BRU-IUL), Lisboa, Portugal

**Keywords:** LGB people, Invariance, Satisfaction with Life Scale, Sexual orientation, Well-being

## Abstract

The Satisfaction with Life Scale (SWLS) has shown strong evidence of reliability, validity, and gender invariance, and there is some evidence of invariance across age, culture, and educational levels. So far, invariance across sexual orientation has not been studied, despite the number of works that relate well-being to sexual orientation. The SWLS should be invariant across sexual orientation to be able to compare group means. This study aimed to explore the invariance of the SWLS across sexual orientation. A non-probabilistic sample with 553 Spanish adults (208 males, 345 females; 212 heterosexuals, 182 gays, and 138 bisexuals among other sexual orientations) participated in a survey. We tested a one-factor model using confirmatory factor analysis. We tested the configural, metric, and scalar invariance of the factorial structure of the SWLS across sexual orientation with heterosexual, lesbian/gay, and bisexual groups. According to our results, the Spanish version of the SWLS shows scalar invariance across sexual orientations, allowing a valid comparison between sexual minority and heterosexual people. Moreover, in our sample, lesbian/gay and bisexual participants obtained lower scores in life satisfaction than heterosexual participants. Bisexual people obtained the lowest score in well-being compared with the other groups. Implications related to the importance of checking instrument invariance before comparing mean differences between groups are discussed.

## Introduction

In line with several studies, lesbian, gay, and bisexual (LGB) people experience poorer mental health and well-being compared with heterosexuals (de Graaf et al., 2006; Meyer, 2003; Semlyen et al., 2016). Past research found that such disparities are due to unique social stressors related to the stigmatization of sexual minority identities (Meyer, 2003), which lead to higher levels of depression, anxiety, destructive behaviors, psychological distress, and lower levels of life satisfaction in LGB people (e.g., Cochran et al., 2003; Meyer, 2003; Michaels et al., 2019). For instance, a combined meta-analysis of 12 UK population health surveys (Semlyen et al., 2016) showed that, compared to heterosexuals, lesbian, and gay participants were more likely to show poorer mental health and worse well-being. In the same line, a systematic review (King et al., 2008) showed that LGB people are at higher risk of mental disorders, suicidal intention, and substance abuse. Researchers also showed that disparities exist between LGB groups, demonstrating that bisexual people are more likely to experience mental health problems and self-harm behaviors (e.g., Jorm et al., 2002; King et al., 2008). In fact, bisexual people have to face specific negative attitudes because people perceive them as unstable and are stereotyped to be confused about their sexual orientation (Dodge et al., 2016; Kuyper & Fokkama, 2011; Mereish et al., 2017).

The interest in LGB people's well-being has increased in recent decades, in line with the interest in the study and measurement of the general population's well-being, which might be studied from different perspectives. There are approaches from the study of happiness (Fredrickson, 2009), lack of negative emotions (Diener et al., 2010; Watson et al., 1988) or a multicomponent approach (Marsh et al., 2020). One of the most used concepts has been satisfaction with life along with positive emotions (Cohn et al., 2009; Kuppens et al, 2008). The most widely used instrument to measure satisfaction with life has been the "Satisfaction with Life Scale" (SWLS) (Diener et al., 1985), which has shown the strongest psychometric characteristics in numerous investigations worldwide (Emerson et al., 2017). Although several studies have explored well-being disparities among LGB and heterosexual people, few research have assessed instrument measurement invariance, taking it for granted (Periard et al., 2018). This omission might represent a serious limitation, because LGB groups and heterosexuals might interpret instruments differently (Periard et al., 2018).

All adaptations of the SWLS show a one-dimensional structure, but to use the scale to compare groups, it is necessary to demonstrate its measurement invariance. Evidence of measurement invariance is a prerequisite for meaningful comparisons of mean scores between groups or over time. Most researchers (e.g., Emerson et al., 2017) typically use multigroup confirmatory factor analysis (MG-CFA) to test for measurement invariance using a 4-level hierarchical model with increasing levels of constraint. There are four levels of invariance: configural, metric, scalar, and strict (Emerson et al., 2017). Configural invariance requires an equivalent factor structure and means that participants use the same conceptual framework to answer. Metric invariance implies equal factor loadings for similar items across groups, and suggests that similar items share equivalent meaning in terms of their relationship to the factor across groups. Scalar invariance implies equivalent intersections and suggests that the probability of endorsing a response (category) for similar items is equivalent across groups. Finally, strict invariance requires equality of residual variances and indicates that the systematic measurement error related to group membership is equivalent across groups for similar items. Each of the levels is nested in the configural model and the invariance of a level must be confirmed to increase the level of constraint. The current literature accepts that the scalar invariance is sufficient to compare latent factor means (Van de Schoot et al., 2012; Vandenberg & Lance, 2000). However, a recent review explained that the SWLS has been used to compare variables between groups in which the scale has not shown strict or scalar invariance, a practice that should not be accepted (Emerson et al., 2017).

Invariance across gender in the SWLS has been tested in some studies (Emerson et al., 2017). Most studies have shown a strict invariance (Bai et al., 2011; Clench-Aas et al., 2011; Moksnes et al., 2014; Shevlin et al., 1998; Tomás et al., 2015) or scalar (Checa et al., 2019; Hultell & Gustavsson, 2008; Jovanovic, 2016; Zanon et al., 2014), that is, that comparisons can be made between the scores of men and women. On another hand, some studies have shown strict (Hinz et al., 2018; Tomás et al., 2015) or scalar (Checa et al., 2019) invariance across age. Few studies have tested invariance across other variables like educational level or marital status (Bai et al., 2011; Checa et al., 2019).

To our knowledge, there is no previous study that tested measurement invariance across sexual orientation. Although some studies (Michaels et al., 2019; Petrou & Lemke, 2018) have already explored the satisfaction with life of LGB people, we do not know previous study that has analyzed the invariance of the SWLS in the comparison of LGB and heterosexual people. For this purpose, it is necessary to verify that the scales that measure subjective well-being (i.e., the SWLS) and that were validated with the heterosexual population, are equally valid for measuring well-being in the LGB population.

The aim of this paper is to explore configural, metric, and scalar invariance of the Spanish version of the SWLS across sexual orientations: heterosexual, lesbian, gay, and bisexual and to replicate and actualize possible differences in subjective well-being.

## Method

### Participants

This study involved 553 people between 18 and 74 years of age (*M* = 31.33, *SD* = 12.03). The sample comprises 208 (37.6%) men and 345 (62.4%) women. According to sexual orientation, 212 people self-identified as heterosexuals (38.3%), 182 (32.9%) as gay people (96 gay men and 86 lesbians), and 138 as bisexual people (25%). Twenty-one participants chose the option "Other sexual orientation" (3.8%). Due to the great difference between the first three sample groups and the last one, the group of "others" was not included for the invariance analysis. Regarding the educational level, n = 45 declared having basic studies, n = 187 secondary studies and n = 321 university studies.

### Measures

The SWLS (Diener et al., 1985) was used to assess satisfaction with life in the version translated and adapted to Spanish (Atienza et al., 2003). The test consists of a 5-item scale designed to measure people’s global cognitive judgments of life satisfaction (e.g., 1. In most aspects, my life is as I want it to be; 2. The circumstances of my life are very good). In the original version, participants indicate their degree of agreement/disagreement with each of the five items on a seven-point scale but the adaptation to Spanish uses a five-point Likert scale ranging from 1 (*strongly disagree*) to 5 (*strongly agree*). The Spanish version obtained excellent psychometric results that demonstrate its factorial structure and reliability (Vazquez et al., 2013).

Besides other sociodemographic variables such as age, gender, and educational level, sexual orientation was obtained by asking people to self-identify their sexual orientation through the question “What is your sexual orientation?”, with four response options: heterosexual, homosexual (gay men and lesbian), bisexual and others. When the response was “others,” participants were invited to specify their sexual orientation.

### Procedure

A triple strategy was organized for data collection, the same for the different sexual orientations that participated in the study. Firstly, we contacted two LGBT rights organizations (Triangle Foundation and Andalusian Rainbow Federation), which disseminated the link of the online questionnaire on their social networks. Secondly, we published the information about the study on several forums related to LGBT movements and requested participation through the online questionnaire. Finally, we requested the participation of LGBT students enrolled in different courses at the University of Huelva; students could choose to fill in the online questionnaire or the paper-and-pencil questionnaire. For any of these strategies, anyone who wanted to participate could do so regardless of sexual orientation.

All participants had to be over 18 years of age and were given informed consent to be signed and accepted. In the online procedure, they had to accept the consent form to access the Online Form; otherwise, it did not open.

### Data Analysis

We tested the one-dimensional structure that supports the literature (Emerson et al., 2017) and performed a confirmatory factor analysis (CFA). For this, we used Mplus 8.4 (Muthén & Muthén, 1998–2017) and chose the MLR estimator (maximum likelihood robust) to estimate the parameters and statistics of the model. This estimator was chosen for three reasons: most of the invariance studies that use the SWLS have treated the data as continuous (Emerson et al., 2017), MLR is considered the best estimator with non-normal data and Likert scales of five or more points (Finney & DiStefano, 2006). We calculate chi-square (χ^2^), the comparative adjustment index (CFI) and the mean squared error of approximation (RMSEA) with 90% confidence interval to evaluate the goodness of fit of the proposed models. The cutoff points for goodness of fit were 0.90 / 0.95 for CFI (acceptable / excellent) and 0.08 / 0.06 (acceptable / excellent) for RMSEA (Chen, 2007).

Three nested models with an increasing degree of restriction were tested. First, the base model allowed the free estimation of all the parameters for each group (configural invariance). Secondly, the metric invariance model, nested in the base model, added the restriction of invariant factor loads between groups. Finally, nested in the second model, the intercept restriction of the invariant elements in the comparison groups was added (scalar invariance model). The CFI and RMSEA indices were mainly used in the comparison, since the chi-square indices are sensitive to the sample size. We followed Chen’s (2007) recommendations for non-invariance (ΔCFI $\ge .$ 01, supplemented by ΔRMSEA $\ge$0.015), and Hirschfeld and von Brachel’s (2014) or Hu and Bentler’s (1999) suggestions that ΔCFI is the best indicator (when ΔCFI < 0.01). To evaluate the normality of the distributions, the symmetry and kurtosis were calculated for each item. We also assessed the composite reliability of the scale using Raykov’s coefficient, considering values to be acceptable if they reached 0.70 (Raykov, 1997), in addition to the traditional Cronbach's alpha coefficient.

## Results

First, descriptive SWLS data were calculated for the complete sample and for the heterosexual, gay people (lesbians and gay men), and bisexual groups (Table 1). There are no missing data. Regarding the normality of the data, the items present an asymmetry with a range between -0.22 and -1.01 and kurtosis with a range between -0.92 and 0.83, from which it follows that the distribution of the data should be considered as non-normal. The composite reliability (Raykov coefficient) and Cronbach´s alfa were good with a value of 0.89 and 0.88, respectively. Table 1 shows the Cronbach's alpha values ​​for the three groups separately and all are good. The one-dimensional model for the complete sample presented an excellent fit, χ^2^ = 6.70 (*df* = *5*); CFI = 0.998; RMSEA = 0.025. The standardized factor loadings present values ​​greater than. 400 (It1 = 0.807; It2 = 0.810; It3 = 0.904; It4 = 0.770; It5 = 0.617).

**Table 1:** Item means and standard deviations for the total sample and all subgroups

		I1		I2		I3		I4		I5		Total		α
		Mean	*SD*	Mean	*SD*	Mean	*SD*	Mean	*SD*	Mean	*SD*	Mean	*SD*	
Total Sample		3.21	1.12	3.38	1.06	3.44	1.07	3.59	1.08	2.98	1.27	16.59	4.63	.882
Sexual orientation	Heterosexual people	3.44	1.05	3.61	.95	3.65	.99	3.83	.96	3.19	1.17	17.73	4.19	.875
	LG people	3.15	1.15	3.38	1.08	3.41	1.09	3.62	1.11	2.95	1.33	16.51	4.87	.896
	B people	2.96	1.14	3.10	1.07	3.21	1.09	3.22	1.11	2.77	1.31	15.25	4.57	.853

*SD* = Standard deviation; α = Cronbach’s alpha; Item 1. In most ways, my life is close to my ideal; Item 2. The conditions of my life are excellent; Item 3. I am satisfied with my life; Item 4. So far, I have gotten the important things I want in life; Item 5. If I could live my life over, I would change almost nothing

### Configural, Metric, and Scalar Invariance

Table 2 shows the results of the model through sexual orientation and nested invariance models, in ascending order of level of restriction. The one-factor model shows adequate fit indices in the three separate groups, although the group with the poorest fit is heterosexuals. The model shows a good fit in the restriction of factor loadings (metric invariance) and intercepts (scalar invariance). The results showed that the SWLS had a strong invariance across sexual orientation, and the fit of the one-dimensional model for heterosexuals, gay people (lesbians and gay men), and bisexuals was good, meaning that the probability of supporting a response (category) for equal items is equivalent in all groups. These results allow the comparison of the latent means of each sexual orientation because the ΔCFI between three models of invariance was < 0.01.

**Table 2:** Tested models and goodness-of-fit indices

Models in each group		χ^2^	*df*	Δχ^2^	Δ*df*	CFI	RMSEA (90% CI)	ΔCFI	ΔRMSEA
SO	Heterosexual people	15.407*	5			.963	.099 (.045-.015)		
	LG people	2.603*	5			.999	.002 (.000-.007)		
	B people	6.149*	5			.995	.041 (.000-.013)		
Global model									
Configural		25.765*	15	-	-	.988	.064 (.013 -.104)	-	-
Metric		28.619*	23	2.477	8	.994	.037 (.000-.007)	.006	-.027
Scalar		37.417*	31	10.744	8	.993	.034 (.000-.006)	-.001	-.003

SO = Sexual Orientation

^*^All chi-squares are significant at *p* < .001; *df* = degrees of freedom; χ^2^ = chi-square; Δχ^2^ = difference in chi-square; Δdf = difference in degrees of freedom; CFI = Comparative Fit Index; TLI = Tucker–Lewis Index; RMSEA = Root Mean Square Error of Approximation

### Differences Across Sexual Orientation

After checking the scalar invariance, it is now possible to calculate the differences in the latent means of satisfaction with life between the groups evaluated. The latent mean values were fixed to zero for heterosexuals. Lesbian and gay people had poorer satisfaction than heterosexual (*b* = -0.246, *z* = -2.582, *p* = 0.01) and bisexual people had worse satisfaction than heterosexuals too, with an even larger difference than with lesbian and gay people (*b* = -0.493, *z* = -4.83, *p* < 0.01).

## Discussion

This article aimed to study the invariance of the SWLS to obtain greater psychometric evidence of the Spanish version of this scale and to have an instrument that measures differences in well-being in groups of different sexual orientations. Our findings show that the SWLS can be used to compare heterosexual, gay people (lesbians and gay men), and bisexual people. It is important to check the measurement invariance because conclusions are sometimes expressed without demonstrating that the items that make up a scale evaluate the construct in the same way in all the analyzed groups (Slof-Op’t Landt et al., 2009).

By showing the sexual orientation invariance of the SWLS, we make an important contribution that allows advancing in the research of LGB people's well-being, using a valid instrument. Our data confirmed previous studies on well-being disparities among LGB and heterosexuals (Cochran et al., 2003; Meyer, 2003; Michaels et al., 2019), showing that lesbian, gay, and bisexual participants obtained a lower score in life satisfaction than heterosexual participants. Moreover, our study confirmed that well-being differences exist between minority sexual orientations, being bisexual people more prone to experience mental health problems compared with lesbian and gay people, as previous studies showed (Dodge et al., 2016). In fact, bisexual people have to cope with unique and negative attitudes from heterosexual people as well as lesbian and gay people (Brewster & Moradi, 2010). According to our study and in terms of satisfaction with life, group differences are not due to measurement artifacts, but reflect mean differences between the groups involved.

Findings of LGB people's lower levels of psychological well-being can be explained according to the minority stress conceptual model (Meyer, 2003; Meyer & Frost, 2013) which posits that LGB people are exposed to unique stressors due to negative attitudes and societies’ stigmatization of non-normative sexual orientations. Such stressors can affect LGB people's satisfaction with life considerably, with an important impact on their psychological well-being. Recent studies (Michaels et al., 2019; Petrou & Lemke, 2018) showed that distal stressors (e.g., discrimination) have a direct or indirect impact (through internalized homophobia) on LGB people's satisfaction with life. Internalized homophobia (a proximal stressor) also has a direct impact on life satisfaction.

Although several previous studies (Michaels et al., 2019; Petrou & Lemke, 2018; Rieger & Savin-Williams, 2012) have focused on the relationship between stressors experienced by minority sexual orientations and their satisfaction with life, they did not test the measurement invariance of the SWLS between heterosexual and gay, lesbian and bisexual people to ensure that the instrument performs the same with the groups it compares and therefore that its results are not affected by a measurement artifact. This study overcomes this gap.

This study presents some limitations. Firstly, the sample is not representative of the Spanish population, as it used non-probabilistic sampling. Moreover, our participants were mostly recruited through LGBT rights organizations and social networks. Thus, our findings might have been biased by the recruitment process, because participants might share previous similar experiences. Additionally, most of participants were well-educated. Previous studies (Huynh et al., 2020) have showed that people with higher education might live in more liberal and less discriminatory environments. Therefore, it is not possible to generalize our findings. Additionally, the recruitment process did not allow to calculate the response rate, because we do not know how many people had access to the survey. Secondly, the size of the sample of people who did not self-identify as lesbians, gays, or bisexuals (e.g., “emerging identities,” such as demisexuals, pansexuals, and polysexuals) (Borgogna et al., 2019) did not allow testing invariance in all the subgroups identified. Future research should overcome this limitation, trying to collect data from people belonging to emerging identities to test the measurement invariance of the SWLS. In fact, previous studies showed that they experience higher levels of mental health problems compared with LGB people (e.g., Borgogna et al., 2019), but again we do not know if it is due to measurement artifacts or real group differences. Thirdly, our article assesses sexual orientation by using a single question with limited response options; although the response option "Others" was included for those people who did not self-identify with the categories proposed, more nuanced instruments could have been applied. Finally, intersectionality (Crenshaw, 1989) could not be verified for the participants of this study. Therefore, we could not assess measurement invariance taking into account the intersection of multiple stigmatized identities (e.g., gender identity, and ethnicity). Future research should address this gap.

Despite these limitations, the main contribution of this study is that it shows that the SWLS is also valid for LGB people. One of the main implications of this research is to validate previous studies that have applied the SWLS to analyze disparities between LGB people and heterosexuals, at least when used with Spanish population. Moreover, having a valid and invariant instrument to measure satisfaction with life gives confidence to those practitioners that assess this variable as an important aspect of LGB people's well-being. To sum up, this information is an important first step for researchers and practitioners who want to improve, and further their knowledge in the field of LGB people's well-being by exploring their satisfaction with life.
